# Identifying key policy objectives for strong primary care: a cross-sectional study

**DOI:** 10.1017/S1463423623000403

**Published:** 2023-08-14

**Authors:** Sarah Burgmann, Muna Paier-Abuzahra, Martin Sprenger, Alexander Avian, Andrea Siebenhofer

**Affiliations:** 1 Institute of General Practice and Evidence-based Health Services Research, Medical University of Graz, Neue Stiftingtalstraße 6, Graz 8010, Austria; 2 Institute of Social Medicine and Epidemiology, Medical University of Graz, Neue Stiftingtalstraße 6, Graz 8010, Austria; 3 Institute for Medical Informatics, Statistics and Documentation, Medical University of Graz, Neue Stiftingtalstraße 6, Graz 8010, Austria; 4 Institute of General Practice, Johann Wolfgang Goethe University Frankfurt, Frankfurt 60590, Germany

**Keywords:** health care reform, health policy, health priorities, primary care, questionnaire, stakeholder participation

## Abstract

**Aim:**

The aim of this study was to identify key policy objectives by investigating the perception of important stakeholders and affected professionals concerning relevance and feasibility of a successful primary care (PC) reform.

**Background:**

Since 2013, the Austrian PC system has been undergoing a reform process to establish multiprofessional primary care units. The reforms have various defined objectives and lack clear priorities.

**Methods:**

After the definition and consensus-based selection of 12 policy objectives, a cross-sectional online survey on their relevance and feasibility was distributed via email and social media to PC and public health networks. The survey was conducted in the period from January to February 2020. Results were analyzed descriptively, and further, Pearson Chi-Square Test or Fisher’s Exact Test was performed for group comparison regarding respondents’ characteristics. Open-ended responses were analyzed using qualitative content analysis.

**Findings:**

In total, 169 questionnaires were completed. A total of 46.3% of the responders had more than 20 years of professional experience (female: 60.5%). A mandatory internship in general practice, vocational training for general practice, and a modern remuneration system were the three top-rated policy objectives regarding relevance. A mandatory internship in general practice, specialization in general practice, and coding of services and diagnosis were assessed as the most feasible objectives. The group comparisons regarding working field, years of professional experience, age, and sex did not show any meaningful results in the evaluation of relevance and feasibility.

**Discussion:**

In the view of the study participants, easily obtainable objectives include adapting the duration and setting of internships for medical students, as well as mandatory vocational training for GP trainees. Further efforts are necessary to achieve complex objectives such as the adoption of a modern remuneration scheme and a comprehensive quality assurance program. Building capacity and creating team-oriented environments are also important aspects of a successful PC reform.

## Introduction

A strong primary care (PC) sector can contribute to strengthening the overall health system’s performance by providing affordable, accessible, and coordinated care and by reducing avoidable hospital admissions (Kringos *et al.*, 2019). This was recently reemphasized by a Policy Brief of the Organization for Economic Co-Operation and Development (OECD) on how to realize the full potential of primary health care (PHC) (OECD, 2020b). It is described as a non-static concept, which should be regularly adapted on occurring challenges regarding technology, demographic, and epidemiological trends as well as paths and organization within the health system (European Commission, 2014).

The Austrian health care system is complex and fragmented. Responsibilities for planning and financing are shared between the nine federal states, the social health insurance (SHI), and the federal government. Federal states are predominantly responsible for inpatient care, financed primarily through taxes. SHI is responsible for out-of-hospital care and PC, financed primarily through social security contributions (Bachner *et al.*, 2018).

PC in Austria is mainly provided by self-employed general practitioners (GPs) in single-handed practices (Hoffmann *et al.*, 2015; Bachner *et al.*, 2018), as is the case in half of European countries (Kringos *et al.*, 2015). In 2016, multiprofessional primary care units (PCUs) started to evolve as efforts to strengthen PC within the health care reform – an important step to further shift activities out of the large and costly hospital sector and improve the skill mix within the health workforce. Corresponding to this reform process, the expert panel on effective ways of investing in health (EXPH) of the European Commission states the need of interprofessional care teams and networks within the PC sector to tackle demographic and socioeconomic challenges, for example, multimorbidity, and provide integrated care (European Commission, 2014).

The remuneration system in PC is a mix of contact capitation and fee-for-service remuneration with a focus on the second, which incentivizes high frequencies with low consultation times (Czypionka *et al.*, 2015; Redaèlli *et al.*, 2015; Bachner *et al.*, 2018). Although payment models influence the attractiveness and quality of PC (OECD, 2020b) as well as team-based care (Freund *et al.*, 2015), only 13 OECD countries offered innovative payment models in 2018 (OECD, 2020b).

The reform will be challenging to implement. In the near future, a sufficient number of GPs cannot be guaranteed because too few young doctors are specializing in general practice (Stigler, 2020). The OECD has observed the same situation in several countries because of the lower remuneration and reputation of GPs compared to specialists of other disciplines (OECD, 2021). Vocational training for GPs in Austria is carried out mainly in the inpatient setting with only 6 months in PC and no specialist title being awarded. In 2020, there were four departments for general practice in Austria, but they are not involved in postgraduate training. By contrast, “Huisarts” in training in the Netherlands spend 24 months in PC and one day per week at university (Sönnichsen and De La Cruz Gomez Pellin, 2020). With an average of 44.5 GPs per 100 000 population (Österreichische Ärztekammer, 2020), Austria is clearly below the European average of 68 GPs per 100 000 (Kringos *et al.*, 2015).

An overall nursing shortage and the lack of appealing conditions for nurses in PC is another challenge to successful reform (Rappold and Juraszovich, 2019). In order to provide well-coordinated and accessible interprofessional PC, tasks may be delegated from GPs to other health professionals, especially nurses (European Commission, 2014). For Austria, no data are available on the number of nurses working in PC (OECD and European Union, 2020), compared to around 24 nurses per 100 000 (absolute: 16 040) nurses working in PC in the United Kingdom in 2018, with a rising trend in advanced practice nurses (APNs) (rise of 8.2% from 2017 to 2018) (The Health Foundation, 2019).

PC policy objectives lack concrete priorities, even though clear priorities could be seen as crucial to successful reform. Little is known about the perception of different PC stakeholders (health care providers, research, policymakers and public administration) regarding the setting of priorities on PC policy objectives in Austria.

Therefore, the research purpose of the present study was to investigate the perceptions of health care providers, researchers, administrators, and policymakers on policy objectives, which are essential for a successful reform of PC. Based on these insights, the aim of the study was to identify key policy objectives for strong PC in Austria involving the stakeholders’ perceptions on relevance and feasibility.

## Materials and methods

To respond to the research aim of the present study, a mixed-method design was chosen. This comprises, in the first step, a consensus process within a focused document analysis by the authors and, in the second step, a cross-sectional online survey.

### Questionnaire development

The policy objectives, which are relevant for strong PC and a successful reform, were formulated based on key Austrian (Rabady *et al.*, 2018; Sönnichsen and De La Cruz Gomez Pellin, 2020; BKA, 2020; BMG, 2017) and international reports (WONCA, 2013; Schäfer *et al.*, 2011; Velasco Garrido *et al.*, 2011; Ghebrehiwet, 2013; Kralj and Kantarevic, 2013; Freund *et al.*, 2015; Kringos *et al.*, 2015; OECD/EU, 2016; Srivastava *et al.*, 2016; OECD and European Union, 2018) on essential PC aspects. The Primary Care Assessment Tools (Shi *et al.*, 2001) and the PHAMEU study (Kringos *et al.*, 2010) served as an orientation. The objectives were selected and formulated based on a consensus process by the authors with a 10-year period from 2020 to 2030 being set as the period for achieving the objectives. Table 1 shows the short titles of the final objectives. For more details, additional file 1 shows the resulting objectives and their status quo in Austria.

### Structure of the questionnaire and survey

For the cross-sectional online survey, the platform “SurveyMonkey®” was used. The questionnaire consisted of 36 items concerning 12 objectives. For each of the 12 objectives, the participants assessed the relevance of target achievement and the perception of the difficulty of achieving that policy objective (feasibility) using a single choice option. Additionally, the respondents were able to add comments and suggestions to each objective (open-ended questions). Personal and professional information was assessed by five items (occupation, age, education, number of years in the profession and in the current activity).

Closed questions were based on a Likert-type response scale in the range of one to five where one was “very important”/“very easy” and five was “very unimportant”/“very difficult.” A pilot web questionnaire was sent to five individuals working at a PCU, a university, a SHI provider, and a GP practice comprising the professions physical therapist, dietician, nurse, GP, and psychologist to test the comprehensibility and technical functionality of the questionnaire. The online questionnaire was further refined after the responses from this pilot survey to make it more readily understandable and easier to answer.

The data are stored on the server of the Institute for General Medicine and Evidence-based Health Services Research, Medical University of Graz, and are only accessible via registration data.

### Target group, recruitment process, and survey administration

The target group of this study was persons, who are familiar with the topics of public health and/or PC to ensure a basic knowledge of PC in Austria.

In order to reach representatives from the micro- (intra- and extramural practice), meso- (research and representatives of professional representation, lobbyists), and macro- (policy and public authorities) levels (Smith *et al.*, 2019), convenience sampling for this open survey was performed (Clark, 2017). The questionnaire was distributed via pertinent Google groups as well as Facebook groups, two mailing lists, and an Instagram profile. In total, the web survey (baseline) was sent to 1,412 mail contacts via two Google groups (Public Health, *n* = 480 and PC *n* = 409) and two mailing lists (Public Health School Graz, *n* = 488, and the core team of the Association of Young GPs in Austria, *n* = 35) at the beginning of January 2020. The survey was also promoted via social media channels with a total of 703 followers comprising the Facebook profiles of the Public Health School Graz (followers: *n* = 419) and the Austrian Forum for PC (followers: *n* = 271) as well as the Instagram profile of the Austrian Forum for PC (followers: *n* = 13). Because of multiple mailing lists and sharing via social media, some recipients may have been reached multiple times and a response rate could not be calculated. There were no incentives for participation. The participants were informed about the planned publication of the results at the beginning of the survey. The online survey started on 8 January 2020 and ended on 3 February 2020.

### Statistical analysis

Descriptive statistics were calculated using SPSS 26.0 software (SPSS, Chicago, IL, USA). Only fully completed questionnaires were evaluated. Responses are given as percentages of total responses.

For group comparisons, the response categories “very important” and “important” were collapsed and the other three categories were collapsed indicating “important objectives” versus “not important objectives.” The response categories “easy” and “very easy” were also collapsed and compared to the three other response categories. To compare groups regarding working field, years in profession, age groups, and sex, the Pearson Chi-Square Test or Fisher’s Exact Test was performed.

### Qualitative analysis

Open-ended responses were analyzed using content analysis with the online program “QCAmap.” Content analysis involves identifying, coding, and categorizing the primary patterns that emerge from the collected data. The first step in the process of analysis was to paraphrase comments per objective having reviewed them several times. The second step was adoption of a first draft of the category system. The third step entailed a consecutive reduction by clustering. Finally, a category system was developed by building umbrella categories for the specific sub-categories (Gläser and Laudel, 2009; Mayring, 2015). The results section lists the most frequent topics per objective.

## Results

### Study population

At the end of data acquisition phase of the online survey, 209 questionnaires had been filled out and 169 questionnaires had been completed. Most participants reported that they work in the field of science and research (*n* = 43, 28.1%) or in PC practices (single-handed or group practices or PCU, *n* = 38, 24.8%). Nearly half of the participants (*n* = 76, 46.3%) were experienced health professionals who have been working for more than 20 years, one-third of the respondents were aged between 50 and 59 years (*n* = 49, 30.2%), and two-thirds (*n* = 95, 60.5%) of the participants were female (Additional file 2).

### Relevance of PC policy objective achievement

**Figure 1.**
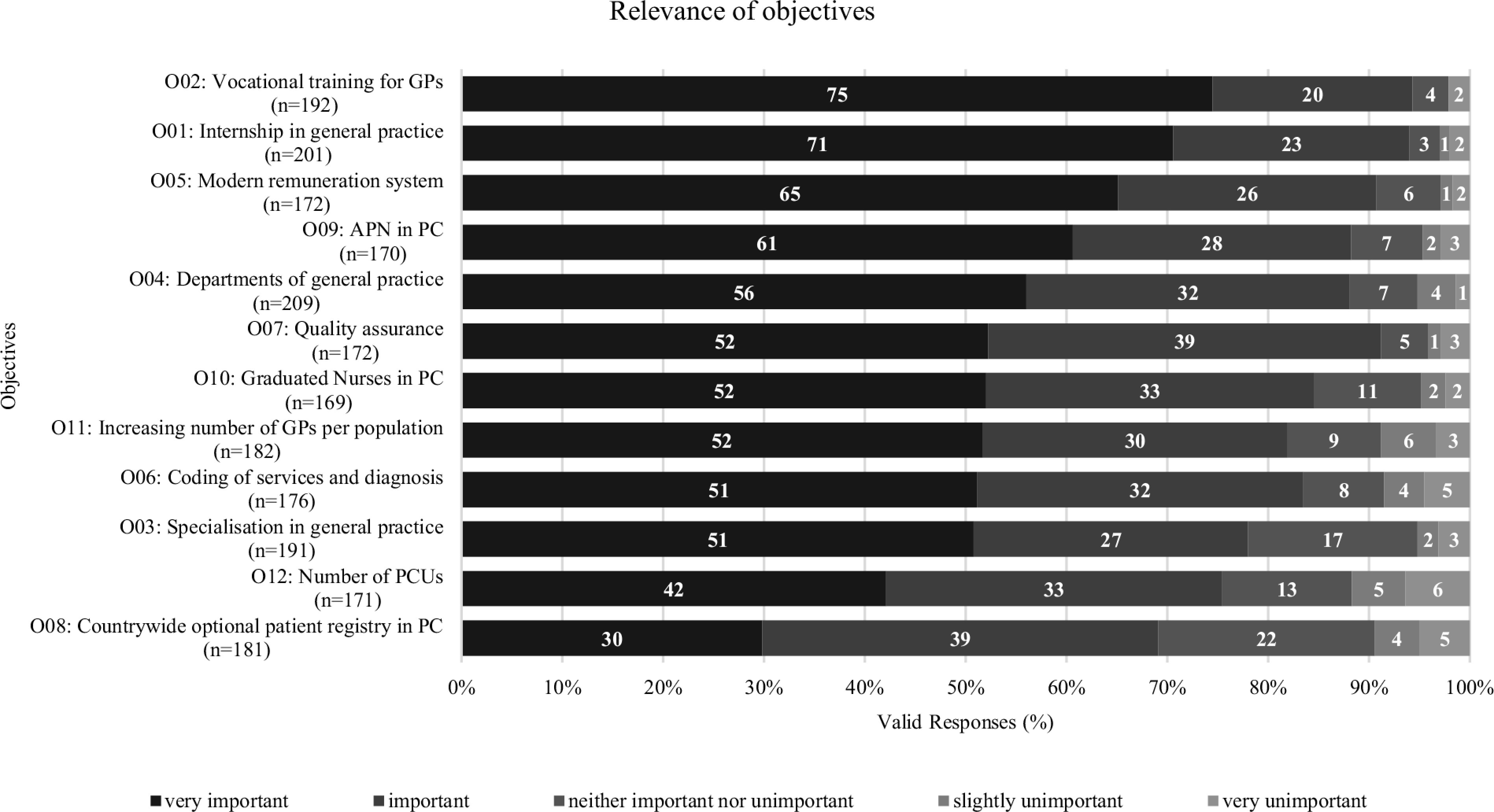
Relevance of objectives. GP = general practitioner; PC = primary care

Figure 1 shows the responses for the 12 objectives regarding the variable relevance

Each objective was rated by at least two-thirds of the respondents as “important” or “very important.” More than 90% of the respondents rated objective 02 “Vocational training for general practice” (95%) and objective 01 “Internship in general practice” (94%) as “very important” or “important.” Also, a very high number of respondents rated objective 05 “Modern remuneration system” (91%) as one of the three most relevant policy objectives. Less than 80% of the respondents rated objective 03 “Specialization in general practice” (78%), objective 12 “Number of PCUs” (75%), and objective 08 “Countrywide optional patient registry in PC” (69%) as being relevant (see Figure 1).

### Feasibility of PC policy objective achievement

**Figure 2.**
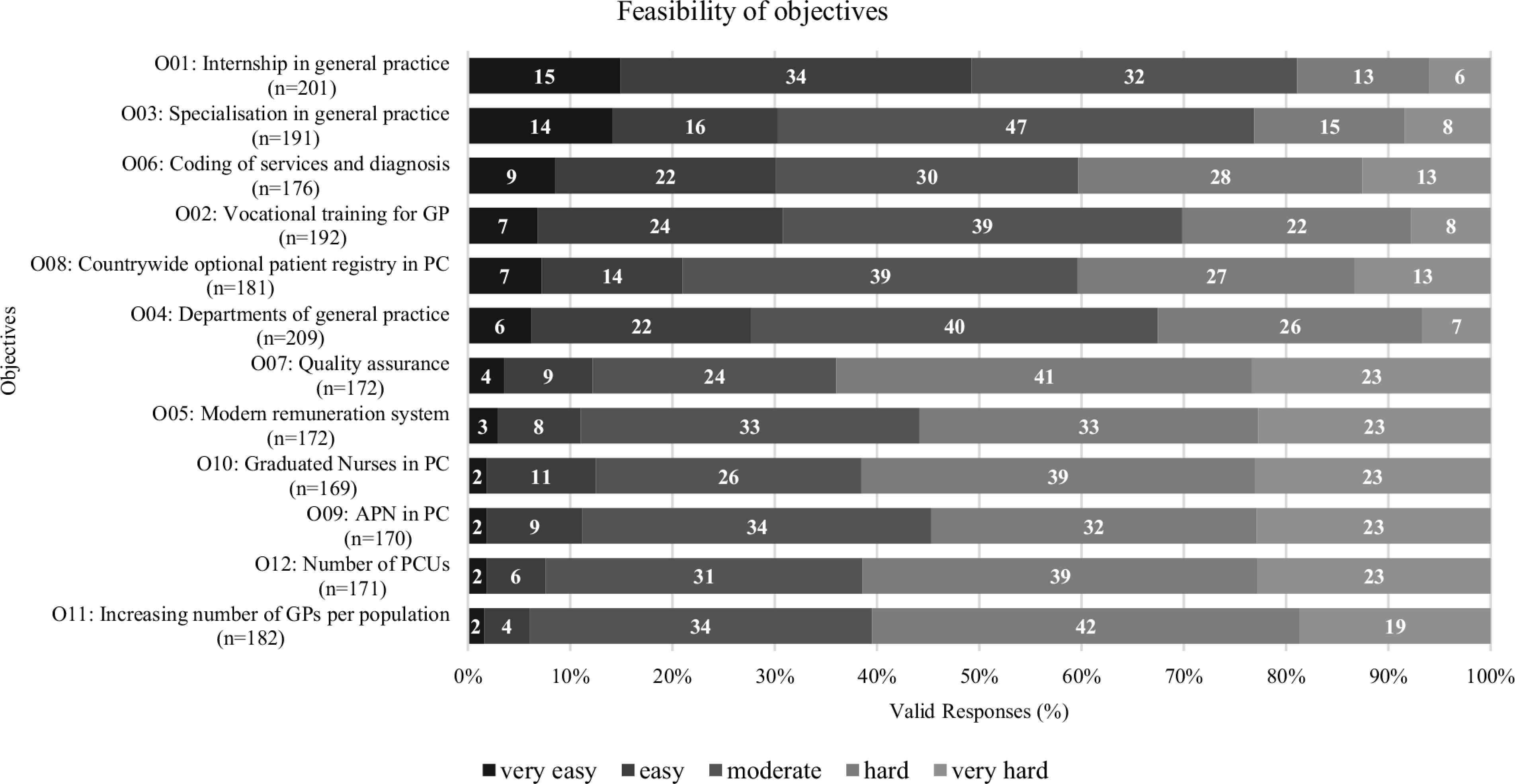
Feasibility of objectives. GP = general practitioner; PC = primary care

Figure 2 shows the responses for the twelve objectives regarding the variable feasibility.

The rated feasibility of the objectives varies considerably.

Objectives 01 “Mandatory internship in general practice” (49%), 03 “Specialization in general practice” (30%), and 06 “Coding of services and diagnosis” (31%) were assessed as the most feasible objectives by the participants, being mostly considered “very easy” and “easy.” The objectives 07 “Quality assurance” (74%), 10 “Graduated Nurses in PC” (62%), and 12 “Number of PCUs” (62%) were rated as the least feasible, receiving the most ratings as being “very hard” and “hard.”

**Figure 3.**
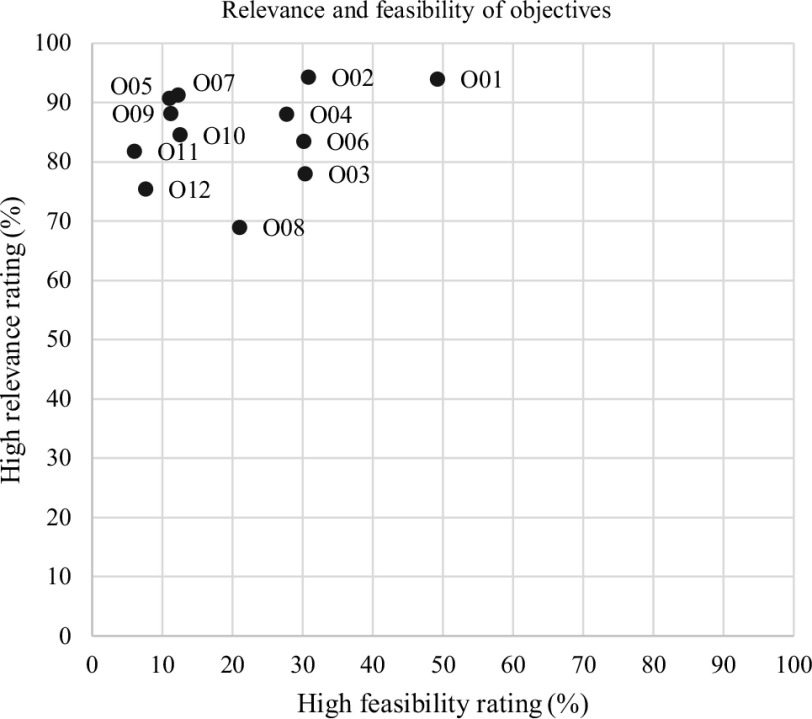
Relevance and feasibility of objectives

Figure 3 depicts the proportion of objectives that were rated as “very easy” or “easy” to achieve and “very important” or “important,” respectively, and mapped to each other. In general, this figure shows that almost all objectives were assessed as highly relevant by a high number of respondents (> 70%). In contrast to the rating of relevance, the feasibility rating shows a higher variation and did not get as many positive responses. However, objective 01 “Internship in general practice” was considered the most important and feasible. In addition, the results for objective 02 “Vocational training for GPs” show high levels of reported relevance and feasibility. In contrast, objective 08 “Countrywide optional patient registry in PC” was assessed by the fewest respondents as being highly relevant but was seen as moderately feasible.

### Comparison of groups

The group comparisons regarding working field, years of profession, age, and sex did not show systematic differences in the evaluation of relevance and feasibility. There were some sporadic significant results (Additional file 3).

We found no differences in terms of age. Sex had an influence on the assessment of the relevance of “graduated nurses in PC” (objective 01), “vocational training for general practice” (objective 02), as well as on the assessment of feasibility in terms of a “modern remuneration system” (objective 05). More women consider “vocational training” to be (very) important (female: 98.9% versus male 90.3%; Fisher’s Exact *P* = 0.016) and “graduated nurses in PC” to be (very) important (female: 84.2% versus male 66.1%; Pearson Chi-Square *P* = 0.008). Women are less likely to rate the feasibility of a “modern remuneration system” as easy (female: 2.1% versus male 11.3%; Fisher’s Exact *P* = 0.029). Those with more than 20 years’ professional experience are more likely to rate the importance of “Specialization in general practice” (objective 03) as important (> 20 years: 100%, 11–20 years: 88.1%, < 10 years: 90,7%; Fisher’s Exact *P* = 0.004). The field of working also had an impact on the evaluation of two objectives, “quality assurance” (objective 07) and “graduated nurses in PC” (objective 10). Those working in science and research are more likely to rate “quality assurance” and “graduated nurses in PC” as important than those working in other fields, for example, ambulatory care (science and research 95.3%, inpatient care 90.9%, public administration 87.9%, ambulatory care 71.1%, associations, and nonprofit organizations 70.0%; Fisher’s Exact *P* = 0.012, respectively; science and research 86.0%, inpatient care 81.8%, associations and NPOs 80.0%, public administration 78.8%, ambulatory care 57.8%; Pearson Chi-Square *P* = 0.028).

### Qualitative analysis of comments

The number of comments per objective ranged from 18 to 31 (median: 27). The proportion of respondents per objective ranged from 9% to 19% (median: 12%). Regarding the number of comments per respondent, results show that a maximum of 7 respondents made 11 comments and 134 respondents made no comments.

#### Objective 01 “Internship in general practice” + 02 “Vocational training for general practice”

Those commenting on objectives 1 and 2 selected lack of relevance of implementation for stakeholders as well as minimal availability of traineeships within PCUs as central themes. The main factor reported as a reason for the absence of traineeships is the general lack of GPs.

Acting as a supervisor for medical students alongside day-to-day practice regarding objective 01 means additional time and effort for trainers, but no financial allowance is provided either for them or for medical students, unlike internships in a clinical setting. Respondents also mentioned that early exposure to the discipline of general practice could have a positive impact on the choice to specialize in general practice. Regarding vocational training, respondents complained about lower levels of income for vocational training in general practice settings in contrast to training in clinical settings. Further comments dealt with the importance of learning from experienced GPs in PC settings and expanded on the duration and valorization of general practice as a specialty.

#### Objective 03 “Specialization in general practice”

Many comments recommended the implementation of general practice as a specialty in Austria to improve its reputation in comparison with other disciplines. Among the suggested required measures were the specialty definition and regulations linked with a specialist title, adaptation of vocational training, and support by medical association. Respondents justified their rating as feasible and with suggestion of a soon implementation by definition of objective 03 as a policy target in the current government program.

#### Objective 04 “Departments of General Practice”

Regarding objective 04, respondents mainly reported lack of relevance for stakeholders, federalism, and lack of financial resources as impediments to achieving objectives. The qualification of medical personnel as experienced GPs and the positive effect on the reputation of the specialty of general practice were also mentioned.

#### Objective 05 “Modern remuneration system”

Respondents commented critically regarding the implementation of a uniform remuneration system because of the many stakeholders involved and their disagreements. The merging of the regional health insurance fund into an Austrian health insurance fund was mentioned as potentially beneficial. Respondents also reported the potential of shifting from a quantitative to a qualitative orientation in PC.

#### Objective 06 “Coding of services and diagnosis”

Comments on consistent documentation of services and diagnoses by coding with ICPC-2 show the demand of respondents for practical software as well as training on how to achieve high data quality, reduce effort, and increase the willingness of GPs to use coding. The achievement of objective 06 was commented on as especially relevant, not least for research. Nevertheless, respondents also made critical comments on the coding format of ICPC-2 and its relevance in practice and mentioned the resistance of the Austrian Medical Chamber.

#### Objective 07 “Quality assurance”

Some respondents criticized the measurement of process and outcome quality indicators, because of perceived lack of relevance and a lack of understanding of stakeholders and GPs on how to use them. External monitoring and meaningful representation of all relevant issues affecting quality in a PC practice were also mentioned by respondents as challenging. Some comments also highlighted the relevance of quality measurement.

#### Objective 08 “Countrywide optional patient registry in PC”

Comments on the patient register highlighted relevance but mentioned structural issues in the PC system, as well as the lack of prioritization for stakeholders and conflicting interests, as the main barriers to implementation. The importance of public information campaigns to foster acceptance and behavioral changes for usage of health system services was reported as precondition for implementation. Two respondents advocated a mandatory patient registry instead of voluntary option.

#### Objective 09 “Advanced practice nurse (APN) in PC”

In comments on objective 09, respondents mainly complained about the APN education structures in Austria. According to respondents, since there is currently no specific regulation of this profession and limited availability of traineeships and a correspondingly reduced number of trained APNs, certain aspects regarding competencies, financing, and the role of the APN in PC are widely unknown. While the relevance of this topic was highlighted by some, others also questioned the need for APNs and worried about competition with GPs.

#### Objective 10 “Graduated Nurses in PC”

The main impediment to increased involvement of graduate nurses in PC (objective 10) is the general shortage of nurses in Austria. The need for adequate pay as well as a cultural change in working conditions was also mentioned.

#### Objective 11 “Increasing number of general practitioners per population”

Comments regarding achievement of objective 11 “Increase of GPs per population in PC” mainly dealt with the shortage of GPs in general, as well as the low attractiveness of the job, especially for young doctors and in rural areas. Additional criticism concerned the need for an increase in the number of GPs. Positive voices highlighted the benefit of having more time per patient. Respondents mentioned the need for policymakers, funders, and the Austrian Medical Chamber to demonstrate willingness and perceive relevance as the main precondition for achieving this objective.

#### Objective 12 “Number of PCUs”

Participants commented regarding the “establishment of 225 PCUs in Austria by 2030” that there is need for a national implementation strategy for PCUs. Respondents reported that the establishment of PC centers and networks is dependent on huge investments by pioneers and should be aimed at regional demands. The current structures are seen as unattractive and policymakers lack ambitious strategies. Critical comments mentioned quality and low-threshold services in the PC system as more relevant than the type of organization.

## Discussion

This cross-sectional study with 169 participants working in the field of public health and PC prioritized major policy objectives based on relevance and feasibility for the Austrian PC reform process.

The study reveals that people who are familiar with the PC and/or public health sector perceive some objectives as highly relevant and relatively easy to put into practice. In particular, mandatory internships in general practice, vocational training for general practice, and university departments for general practice seem to be highly relevant and feasible. In contrast, a modern remuneration system, a quality assurance program, and the establishment of APNs in PC were rated as highly relevant but harder to achieve in terms of implementation.

All the defined and assessed policy objectives are mentioned directly or implicitly in the WHO European PHC Impact, Performance, and Capacity Tool (PHC-IMPACT), which is a tool to monitor the strength and performance of PC systems in Europe (Barbazza *et al.*, 2019). It was published around the time of the survey and, therefore, was not included in the creation of the questionnaire.

### Low-hanging fruits (high relevance and feasibility)

Early introduction of medical students to PC and adequate preparation of future GPs for their tasks are essential. Adapting the duration and setting of internships for medical students, as well as requiring mandatory vocational training for GP trainees, are perceived as relevant “low-hanging fruits” by the study participants and in the literature (Nicholson *et al.*, 2016; Linde *et al.*, 2020). Career paths in Austrian hospitals seem to be more attractive as mentioned by Papp concerning Hungarian pediatricians (Papp *et al.*, 2019). Given that comments on both objectives (internship and vocational training), insufficient remuneration is a relevant hindering factor in different contexts of practical education, so financing might be key for success. Deutsch *et al.* (2019) confirm that acknowledgment of barriers, for example, time restraints, productivity loss, as well as financial issues, could engage more GPs in training. Positive comments on the benefits of juniors learning from seniors concur with the results of Nicholson *et al.* (2016) that GP tutors are inspiring role models. Furthermore, postgraduate vocational training for future GPs, comparable to other disciplines, is essential to raise the reputation of GPs as a first point of contact for all health needs and as the coordinator of the care process (Kringos *et al.*, 2015; WONCA, 2013).

As reported by WONCA and OECD as well as others (Rabady *et al.*, 2018; WONCA, 2013; Sönnichsen and De La Cruz Gomez Pellin, 2020; OECD/EU, 2016), addressing the academic status of health professionals within workforce development strategy is an essential aspect for high-quality health care provision (Kringos *et al.*, 2015). Research in this field enables professionalization, advancement of professions and processes, as well as advocacy for the interests of health professionals (Kringos *et al.*, 2015). The European general practice network provides a guidance to enhance research within the PC community with focus on GPs. They postulate research capacity building as well as improving leadership skills as essential on the micro-, meso-, and macro-level of PC to create a research culture as well as strong collaboration within international networks. Transfer and exchange of the knowledge with users are needed to foster a development process. Furthermore, the discussion of results with users could improve outcomes, for example, by discussing with stakeholders to create policies on PC structures or curricula for the training of PC professionals (Collins, 2022). In accordance with the national and international literature, the participants of this study thought it was highly relevant that PC research be performed at university level by departments for general practice equipped with adequate resources (Kringos *et al.*, 2015, Rabady *et al.*, 2018).

As postulated by policy studies, and also reflected in the respondents’ comments, the introduction of departments for PC is affected by insufficient equipment, funding, and thus also commitment of the relevant stakeholders (Hudson *et al.*, 2019). Training in the community setting is a core element of every medical curriculum and cornerstone for the implementation of multiprofessional and holistic PC (van Weel *et al.*, 2018). These highly relevant and feasible objectives should be considered as “low-hanging fruits” regarding priority setting by policymakers. Furthermore, any investment in skills and competencies is part of capacity building and relevant for sustainable policy implementation as well as the ability to meet future challenges (Hudson *et al.*, 2019). This corresponds with the recommendation of WHO on PHC-goal-driven policy-setting in order to guide investments toward team-based PC, which acts as the hub of the health care system (World Health Organization, 2008). Human resources for health have long been neglected in international health policymaking but are one of the main foundations for other diverse health policies and essential for the development of health systems. A workforce development strategy has to put into account in addition to the number of persons and their qualifications, values (World Health Organization, 2008), and skill mix (World Health Organization, 2017) since they form the PC sector (World Health Organization, 2008).

### Highly relevant efforts (high relevance, low feasibility)

The implementation of a modern remuneration system and a comprehensive quality assurance program, as well as a high number of APNs working in PC, are important objectives. However, due to the low level of feasibility, they require significant effort by multiple stakeholders.

The perceptions of our study participants are in line with the international literature, which suggests that a transparent quality assurance program is needed for every PC system (Kringos *et al.*, 2015; BMG, 2017; Velasco Garrido *et al.*, 2011; Ghebrehiwet, 2013; OECD/EU, 2016). This would require an upgrade of the current self-assessment system. High-quality PC is not only a prerequisite for patient-centered care but could also attract students to choose general practice as a career path (Nicholson *et al.*, 2016).

Reforms should also consider data collection from PC practices to monitor, report, and benchmark the structure, process, and outcome indicators of PC. This could lead to better-informed national health policy-making (WHO, 2018). The experiences of other countries could act as a model of good practice to learn from (Ahmed *et al.*, 2021). Quality assurance has been a delicate topic for health professionals in many countries, so reforms in that area require a particularly transparent and thought-through approach (Waldorff *et al.*, 2016; Chauhan *et al.*, 2017).

Increasing the (financial) attractiveness of the working field PC for professionals and balancing primary and specialty care are main determinants to develop the PC system (Friedberg *et al.*, 2010; Kringos *et al.*, 2015). Remuneration systems in countries with a high-rated PC system often combine different approaches of capitation fee, flat-rate payment, fee-for-service, and/or pay-for-performance (Schäfer *et al.*, 2011; Kralj and Kantarevic, 2013; Kringos *et al.*, 2015; OECD/EU, 2016; Srivastava *et al.*, 2016). The European Commission recommends switching the focus of the reimbursement toward depicting the outputs – even better – outcomes of care rather than the costs occurring of services delivered by the providers, which function as inputs. Ideally, the remuneration system encourages coordinated team-based provision of care over a defined period (European Commission, 2014). Nevertheless, fee-for-service is dominant in the current remuneration system for Austrian GPs and shows the lowest score in the European Primary Care Monitor (Kringos *et al.*, 2010). First, it is a major factor contributing to GPs leaving the public PC system; the perceived inadequacy of remuneration and income security is relevant to the GP workforce shortage (El Koussa *et al.*, 2016). Second, mitigation of the income difference between GPs and specialists could influence the career choice of medical students (Friedberg *et al.*, 2010). Third, the remuneration should address team-based service provision. The fee-for-service scheme in Austria, which mainly reimburses GPs’ tasks, reflects the downward delegation process within teams and, therefore, generates hierarchy and hinders role expansion of professionals other than GPs (Freund *et al.*, 2015; Russell *et al.*, 2018).

Providing nurses with expanded competencies and strengthening their role in PC teams were assessed as highly relevant by the participants and have also been positively implemented in other countries (Owen *et al.*, 2019). Task shifting from GPs to nurses and other professionals could mitigate the workload in respect of the GP workforce shortage (Kringos *et al.*, 2015; OECD and European Union, 2018; Ghebrehiwet, 2013; Freund *et al.*, 2015; Groenewegen *et al.*, 2015; OECD/EU, 2016; OECD, 2020a). Developing multiprofessional PC teams facilitates the evolution of professional roles and increases working field attractiveness. Although accurate data are not available, the number of APNs working in PC may be close to zero in Austria and are generally low in Europe (Freund *et al.*, 2015).

Comments that negate a need for APNs or describe potential competition with the role of GP indicate low awareness of the competencies and benefits of APNs in daily care. Mentioning GPs and APNs as competitors suggests a need for clarification and declaration of the GP and APN roles in PC. Lack of clarity on role, competencies, and financing, as well as the heterogeneity in APN training, impedes their inclusion in multiprofessional PC teams (Currie *et al.*, 2012; Freund *et al.*, 2015). Similar results were found by WHO for community health nurses working in PHC (World Health Organization, 2017). The WHO identifies community health nurses as a key element in the provision of PC, alongside the concept of PHC and the need for their training to reflect practice in care and community settings (World Health Organization, 2017). Numerous studies comment on the need to reduce implementation barriers. New roles and competencies, as well as the benefit for professionals and patients, must be promoted on all levels (stakeholders, managers, and professionals) in order to realize the full potential in practice (Smith *et al.*, 2019). Community health nurses could expand PHC-oriented services such as health promotion, disease prevention, and community orientation. Establishing clear frameworks for practice, promoting collaboration between key stakeholders, improving education and training, and developing advocacy plans to inform the community at large could support professionalization, role clarification, and acceptance of change (World Health Organization, 2017).

### Strengths and limitations

Since the selected policy objectives correspond with the PHC-IMPACT instrument (Barbazza *et al.*, 2019), the scope of policy objectives can be seen as essential for PC reform implementation. So, our questionnaire could serve as a tool for policy objective prioritization and also for other countries, and the defined objectives could be used for decision-making toward PC.

The study aim may be unique, since we found no other study assessing the relevance and feasibility of PC policy objectives for priority setting. The distribution of the online survey was performed within the Austrian public health and PC community and associated networks. As the number of recipients remains unknown, it was not possible to calculate a response rate. Also, biases caused by the convenient sampling method and respondents’ reform experiences cannot be ruled out. One strength of the sample is that almost half of the respondents have more than 20 years of professional experience, which means that these people are probably able to judge feasibility well.

The perceptions of policy objectives regarding team-based PC may differ between professions, based on the example of Irish GPs (Tierney *et al.*, 2016), who were more critical regarding the implementation of PC teams than other professions. This aspect cannot be considered in this study since participants were not asked to identify their profession. This could be another limitation of the study.

**Table 1.** Objectives: numbers and short titles

Nr	Objectives: short title
1	Internship in general practice
2	Vocational training for general practice
3	Specialization in general practice
4	Departments of General Practice
5	Modern remuneration system
6	Coding of services and diagnosis
7	Quality assurance
8	Countrywide optional patient registry in PC
9	Advanced practice nurse (APN) in PC
10	Graduated nurses in PC
11	Increasing number of general practitioners per population
12	Number of PCUs

One weakness of the study could be the period chosen for the objectives, as the PC reform in Austria began nearly 10 years ago. A follow-up assessment after 5–10 years could illustrate the changing attitudes of important stakeholders toward various policy objectives.

## Conclusion

Investment in vocational training for general practice may act as a “low-hanging fruit” to attract young doctors into the PC setting. A well-trained workforce, containing GPs, nurses, and other health professionals, is an essential policy objective to implement multiprofessional PC teams and to foster Austria’s PC reform. Further efforts are necessary to achieve more complex objectives like the implementation of a modern remuneration system, a comprehensive quality assurance program, the establishment of APNs, and well-equipped academic departments for PC. The priorities identified could support policy agenda setting to allow Austria to join the group of countries with a strong PC system.

## Supplementary material

For supplementary material accompanying this paper visit http://doi.org/10.1017/S1463423623000403. click here to view supplementary material
