# Supplementary material for: Identifying key policy objectives for strong primary care: a cross-sectional study
Source: Prim Health Care Res Dev. 2023 Aug 14;24:e52. doi: 10.1017/S1463423623000403 (PMC10466205; doi:10.1017/S1463423623000403)
Supplement: Supplementary file 1 [file phcsup.zip › S1463423623000403sup001.docx]

**Significant results of group comparison**

# Group comparison 1

Collapses of variables

- Field of working

| Questionnaire | Group comparison 1 |
| --- | --- |
| Primary care: single-handed, group practices or PCU | Ambulatory care |
| Community services and secondary care |  |
| Inpatient care (incl. outpatient departments and clinics, long time-care) | *Same* |
| Science and Research | *Same* |
| Public administration (governmental organisations und social health insurance) | *Same* |
| Associations and Non-profit organization | *Same* |

- Age

| Questionnaire | Group comparison 1 |
| --- | --- |
| < 30 years | <40 years |
| 30 - 39 years |  |
| 40 - 49 years | *Same* |
| 50 - 59 years | *Same* |
| 60 - 69 years | 60 years and more |
| ≥ 70 years |  |

- Years in profession:

| Questionnaire | Group comparison 1 |
| --- | --- |
| < 5 years | <10 years |
| 5 - 10 years |  |
| 11 - 20 years | same |
| > 20 years | same |
| no health professional occupation | excluded |

From 96 performed tests six tests were significant.

## Relevance

Collapse of the categories: “very important” and “important” as well as “neither important nor unimportant”, “slightly unimportant”, “very unimportant“

### Objective 2 “Vocational training for general practice”

#### Objective 2 “Vocational training for general practice” * Sex

| **Crosstab** | | | | | |
| --- | --- | --- | --- | --- | --- |
|  | | | Sex | | Total |
|  |  |  | Female | Male |  |
| Objective 2 | neither important nor unimportant,  slightly unimportant,  very unimportant | Count | 1 | 6 | 7 |
|  |  | % Sex | 1,1% | 9,7% | 4,5% |
|  | very important or important | Count | 94 | 56 | 150 |
|  |  | % Sex | 98,9% | 90,3% | 95,5% |
| Total | | Count | 95 | 62 | 157 |
|  |  | % Sex | 100,0% | 100,0% | 100,0% |

| **Chi-Square Tests** | | | | | | |
| --- | --- | --- | --- | --- | --- | --- |
|  | Value |  |  | Exact Sig. (2-sided) |  |  |
| Fisher's Exact Test |  |  |  | ,016 |  |  |
| N of Valid Cases | 157 |  |  |  |  |  |
| a. 2 cells (50,0%) have expected count less than 5. The minimum expected count is 2,76. | | | | | | |
| b. Computed only for a 2x2 table | | | | | | |
| c. The standardized statistic is -2,551. | | | | | | |

### Objective 3 “Specialisation in general practice”

#### Objective 3 “Specialisation in general practice” * Years in profession

| **Crosstab** | | | | | | |
| --- | --- | --- | --- | --- | --- | --- |
|  | | | Years in profession | | | Total |
|  |  |  | > 10 Years | 11-20 Years | >20 Years |  |
| Objective 3 | neither important nor unimportant,  slightly unimportant,  very unimportant | Count | 4 | 5 | 0 | 9 |
|  |  | % Years in profession | 9,3% | 11,9% | 0,0% | 5,6% |
|  | very important or important | Count | 39 | 37 | 76 | 152 |
|  |  | % Years in profession | 90,7% | 88,1% | 100,0% | 94,4% |
| Total | | Count | 43 | 42 | 76 | 161 |
|  |  | % Years in profession | 100,0% | 100,0% | 100,0% | 100,0% |

| **Chi-Square Tests** | | | | | | |
| --- | --- | --- | --- | --- | --- | --- |
|  | Value |  |  | Exact Sig. (2-sided) |  |  |
| Fisher's Exact Test | 10,112 |  |  | ,004 |  |  |
| N of Valid Cases | 161 |  |  |  |  |  |
| a. 3 cells (50,0%) have expected count less than 5. The minimum expected count is 2,35. | | | | | | |
| b. The standardized statistic is 2,394. | | | | | | |

### Objective 7 „ Quality assurance“

#### Objective 7 „ Quality assurance“ * Field of working

| **Crosstab** | | | | | | | | |
| --- | --- | --- | --- | --- | --- | --- | --- | --- |
|  | | | Field of working | | | | | Total |
|  |  |  | Ambulatory care | Inpatient care | Science and Research | Public administration | Associations and Non-profit organization |  |
| Objective 7 | neither important nor unimportant,  slightly unimportant,  very unimportant | Count | 13 | 2 | 2 | 4 | 3 | 24 |
|  |  | % Field of working | 28,9% | 9,1% | 4,7% | 12,1% | 30,0% | 15,7% |
|  | very important or important | Count | 32 | 20 | 41 | 29 | 7 | 129 |
|  |  | % Field of working | 71,1% | 90,9% | 95,3% | 87,9% | 70,0% | 84,3% |
| Total | | Count | 45 | 22 | 43 | 33 | 10 | 153 |
|  |  | % Field of working | 100,0% | 100,0% | 100,0% | 100,0% | 100,0% | 100,0% |

| **Chi-Square Tests** | | | | | | |
| --- | --- | --- | --- | --- | --- | --- |
|  | Value |  |  | Exact Sig. (2-sided) |  |  |
| Fisher's Exact Test | 12,059 |  |  | ,012 |  |  |
| N of Valid Cases | 153 |  |  |  |  |  |
| a. 2 cells (20,0%) have expected count less than 5. The minimum expected count is 1,57. | | | | | | |
| b. The standardized statistic is 1,509. | | | | | | |

### Objective 10 “Graduated Nurses in PC”

#### Objective 10 “Graduated Nurses in PC” * Field of working

| **Crosstab** | | | | | | | | |
| --- | --- | --- | --- | --- | --- | --- | --- | --- |
|  | | | Field of working | | | | | Total |
|  |  |  | Ambulatory care | Inpatient care | Science and Research | Public administration | Associations and Non-profit organization |  |
| Objective 10 | neither important nor unimportant,  slightly unimportant,  very unimportant | Count | 19 | 4 | 6 | 7 | 2 | 38 |
|  |  | % Field of working | 42,2% | 18,2% | 14,0% | 21,2% | 20,0% | 24,8% |
|  | very important or important | Count | 26 | 18 | 37 | 26 | 8 | 115 |
|  |  | % Field of working | 57,8% | 81,8% | 86,0% | 78,8% | 80,0% | 75,2% |
| Total | | Count | 45 | 22 | 43 | 33 | 10 | 153 |
|  |  | % Field of working | 100,0% | 100,0% | 100,0% | 100,0% | 100,0% | 100,0% |

| **Chi-Square Tests** | | | | | | |
| --- | --- | --- | --- | --- | --- | --- |
|  | Value | df | Asymptotic Significance (2-sided) |  |  |  |
| Pearson Chi-Square | 10,894^a^ | 4 | ,028 |  |  |  |
| N of Valid Cases | 153 |  |  |  |  |  |
| a. 1 cells (10,0%) have expected count less than 5. The minimum expected count is 2,48. | | | | | | |
| b. The standardized statistic is 2,374. | | | | | | |

#### Objective 10 “Graduated Nurses in PC” * Sex

| **Crosstab** | | | | | |
| --- | --- | --- | --- | --- | --- |
|  | | | Sex | | Total |
|  |  |  | Female | Male |  |
| Objective 10 | neither important nor unimportant,  slightly unimportant,  very unimportant | Count | 15 | 21 | 36 |
|  |  | % Sex | 15,8% | 33,9% | 22,9% |
|  | very important or important | Count | 80 | 41 | 121 |
|  |  | % Sex | 84,2% | 66,1% | 77,1% |
| Total | | Count | 95 | 62 | 157 |
|  |  | % Sex | 100,0% | 100,0% | 100,0% |

| **Chi-Square Tests** | | | | | | |
| --- | --- | --- | --- | --- | --- | --- |
|  | Value | df | Asymptotic Significance (2-sided) |  |  |  |
| Pearson Chi-Square | 6,941^a^ | 1 | ,008 |  |  |  |
| N of Valid Cases | 157 |  |  |  |  |  |
| a. 0 cells (0,0%) have expected count less than 5. The minimum expected count is 14,22. | | | | | | |
| b. Computed only for a 2x2 table | | | | | | |
| c. The standardized statistic is -2,626. | | | | | | |

## Feasibility

### Objective 5 “Modern remuneration system”

#### Objective 5 “Modern remuneration system” * Sex

| **Crosstab** | | | | | |
| --- | --- | --- | --- | --- | --- |
|  | | | Sex | | Total |
|  |  |  | Female | Male |  |
| Objective 5 | Moderate,  hard,  very hard | Count | 93 | 55 | 148 |
|  |  | % Sex | 97,9% | 88,7% | 94,3% |
|  | very easy,  easy | Count | 2 | 7 | 9 |
|  |  | % Sex | 2,1% | 11,3% | 5,7% |
| Total | | Count | 95 | 62 | 157 |
|  |  | % Sex | 100,0% | 100,0% | 100,0% |

| **Chi-Square Tests** | | | | | | |
| --- | --- | --- | --- | --- | --- | --- |
|  | Value |  |  | Exact Sig. (2-sided) |  |  |
| Fisher's Exact Test |  |  |  | ,029 |  |  |
| N of Valid Cases | 157 |  |  |  |  |  |
| a. 1 cells (25,0%) have expected count less than 5. The minimum expected count is 3,55. | | | | | | |
| b. Computed only for a 2x2 table | | | | | | |
| c. The standardized statistic is 2,412. | | | | | | |

# Group comparison 2

Collapses of variables

- Field of working

| **Questionnaire** | **Group comparison 2** |
| --- | --- |
| Primary care: single-handed, group practices or PCU | Working in practice |
| Community services and secondary care |  |
| Inpatient care (incl. outpatient departments and clinics, long time-care) |  |
| Science and Research | *Same* |
| Public administration (governmental organisations und social health insurance) | *Same* |
| Associations and Non-profit organization | Excluded |

- Years in profession:

| **Questionnaire** | **Group comparison 2** |
| --- | --- |
| < 5 years | 20 years or less |
| 5 - 10 years |  |
| 11 - 20 years |  |
| > 20 years | *Same* |
| no health professional occupation | Excluded |

From 48 performed tests four tests were significant.

## Relevance

### Objective 3 „Specialisation in general practice“

#### Objective 3 „Specialisation in general practice“ * Years in Profession

| **Crosstab** | | | | | |
| --- | --- | --- | --- | --- | --- |
|  | | | Years in profession | | Total |
|  |  |  | 20 Years or less | >20 years |  |
| Objective 3 | neither important nor unimportant,  slightly unimportant,  very unimportant | Count | 9 | 0 | 9 |
|  |  | % Years in profession | 10,6% | 0,0% | 5,6% |
|  | very important or important | Count | 76 | 76 | 152 |
|  |  | % Years in profession | 89,4% | 100,0% | 94,4% |
| Total | | Count | 85 | 76 | 161 |
|  |  | % Years in profession | 100,0% | 100,0% | 100,0% |

| **Chi-Square Tests** | | | | | | |
| --- | --- | --- | --- | --- | --- | --- |
|  | Value |  |  | Exact Sig. (2-sided) |  |  |
| Fisher's Exact Test |  |  |  | ,003 |  |  |
| N of Valid Cases | 161 |  |  |  |  |  |
| a. 2 cells (50,0%) have expected count less than 5. The minimum expected count is 4,25. | | | | | | |
| b. Computed only for a 2x2 table | | | | | | |
| c. The standardized statistic is 2,910. | | | | | | |

### Objective 6 „Coding of services and diagnosis“

#### Objective 6 „Coding of services and diagnosis“ * Field of working

| **Crosstab** | | | | | | |
| --- | --- | --- | --- | --- | --- | --- |
|  | | | Field of working | | | Total |
|  |  |  | Working in practice | Science and Research | Public administration |  |
| Objective 6 | neither important nor unimportant,  slightly unimportant,  very unimportant | Count | 19 | 7 | 14 | 40 |
|  |  | % Field of working | 28,4% | 16,3% | 42,4% | 28,0% |
|  | very important or important | Count | 48 | 36 | 19 | 103 |
|  |  | % Field of working | 71,6% | 83,7% | 57,6% | 72,0% |
| Total | | Count | 67 | 43 | 33 | 143 |
|  |  | % Field of working | 100,0% | 100,0% | 100,0% | 100,0% |

| **Chi-Square Tests** | | | | | | |
| --- | --- | --- | --- | --- | --- | --- |
|  | Value | df | Asymptotic Significance (2-sided) |  |  |  |
| Pearson Chi-Square | 6,344^a^ | 2 | ,042 |  |  |  |
| N of Valid Cases | 143 |  |  |  |  |  |
| a. 0 cells (0,0%) have expected count less than 5. The minimum expected count is 9,23. | | | | | | |
| b. The standardized statistic is -1,044. | | | | | | |

### Objective 7 “Quality assurance”

#### Objective 7 “Quality assurance” * Field of working

| **Crosstab** | | | | | | |
| --- | --- | --- | --- | --- | --- | --- |
|  | | | Field of working | | | Total |
|  |  |  | Working in practice | Science and Research | Public administration |  |
| Objective 7 | neither important nor unimportant,  slightly unimportant,  very unimportant | Count | 15 | 2 | 4 | 21 |
|  |  | % Field of working | 22,4% | 4,7% | 12,1% | 14,7% |
|  | very important or important | Count | 52 | 41 | 29 | 122 |
|  |  | % Field of working | 77,6% | 95,3% | 87,9% | 85,3% |
| Total | | Count | 67 | 43 | 33 | 143 |
|  |  | % Field of working | 100,0% | 100,0% | 100,0% | 100,0% |

| **Chi-Square Tests** | | | | | | |
| --- | --- | --- | --- | --- | --- | --- |
|  | Value | df | Asymptotic Significance (2-sided) |  |  |  |
| Pearson Chi-Square | 6,802^a^ | 2 | ,033 |  |  |  |
| N of Valid Cases | 143 |  |  |  |  |  |
| a. 1 cells (16,7%) have expected count less than 5. The minimum expected count is 4,85. | | | | | | |
| b. The standardized statistic is 1,764. | | | | | | |

### Objective 10 „Graduated Nurses in PC“

#### Objective 10 „Graduated Nurses in PC“ * Field of working

| **Crosstab** | | | | | | |
| --- | --- | --- | --- | --- | --- | --- |
|  | | | Field of working | | | Total |
|  |  |  | Working in practice | Science and Research | Public administration |  |
| Objective 10 | neither important nor unimportant,  slightly unimportant,  very unimportant | Count | 23 | 6 | 7 | 36 |
|  |  | % Field of working | 34,3% | 14,0% | 21,2% | 25,2% |
|  | very important or important | Count | 44 | 37 | 26 | 107 |
|  |  | % Field of working | 65,7% | 86,0% | 78,8% | 74,8% |
| Total | | Count | 67 | 43 | 33 | 143 |
|  |  | % Field of working | 100,0% | 100,0% | 100,0% | 100,0% |

| **Chi-Square Tests** | | | | | | |
| --- | --- | --- | --- | --- | --- | --- |
|  | Value | df | Asymptotic Significance (2-sided) |  |  |  |
| Pearson Chi-Square | 6,130^a^ | 2 | ,047 |  |  |  |
| N of Valid Cases | 143 |  |  |  |  |  |
| a. 0 cells (0,0%) have expected count less than 5. The minimum expected count is 8,31. | | | | | | |
| b. The standardized statistic is 1,782. | | | | | | |
