# Supplementary material for: Identifying key policy objectives for strong primary care: a cross-sectional study
Source: Prim Health Care Res Dev. 2023 Aug 14;24:e52. doi: 10.1017/S1463423623000403 (PMC10466205; doi:10.1017/S1463423623000403)
Supplement: Supplementary file 1 [file phcsup.zip › S1463423623000403sup002.docx]

**Additional file 1**

Table 1 Policy Objectives and status in primary care in Austria: Depiction of the status in primary care corresponding to the policy objectives set in this study. ^^[[1]](#footnote-1)^^

| **Nr** | **PC aspect (short title of the objective)** | **Objective** | **Status in PC in Austria** | **PCAT system characteristics (Starfield et al., 2005)** | **PC features (Kringos et al., 2015)** |
| --- | --- | --- | --- | --- | --- |
| 1 | Internship in general practice | In 2030, all medical students at all public Medical Universities have to complete an internship of at least four weeks in primary care in their final year of study. | It is not mandatory for all medical students to complete an internship in general practice. {Sönnichsen, 2020 #288} | Type of primary care practitioner | Academic status of PC |
| 2 | Vocational training for general practice | In 2030, all future general practitioners have to complete a mandatory 12-month internship in primary care. | Vocational training for general practitioners is carried out mainly in the inpatient setting with only 6 months in PC. By the end of vocational training, general practitioners obtain a generalist title instead of a specialist title {Sönnichsen, 2020 #288}, which is associated with lower remuneration. |  |  |
| 3 | Specialisation in general practice | In 2030, all medical doctors obtain a specialist title for general practice after completion of their vocational training. | By the end of the vocational training, general practitioners obtain a generalist title instead of a specialist title. {Sönnichsen, 2020 #288} |  |  |
| 4 | Departments of General Practice | In 2030, all Austrian public Medical Universities (n=4) have a department for General Practice with at least one full-time equivalent professor and 10 full-time equivalents of academic staff. | In 2020, there were four departments for general practice established at Austrian Universities. {Sönnichsen, 2020 #288}  In 2022, there are 6 medical universities in Austria (Vienna, Graz, Salzburg, Krems, Innsbruck, Linz) with academic departments for general practice. The number of employees ranges from 0 to 16 researchers and lecturers {Johannes Kepler Universität Linz, 2022 #291}{Karl Landsteiner Privatuniversität für Gesundheitswissenschaften GmbH, 2022 #290;Medizinische Universität Graz, 2022 #289;Medizinische Universität Innsbruck, 2022 #292;Medizinische Universität Wien, 2022 #294;Paracelsus Medizinische Privatuniversität, 2022 #293}. | Academic departments of family medicine |  |
| 5 | Modern remuneration system | In 2030, a modern and attractive remuneration system is implemented in Austrian primary care. | The remuneration system in PC is a mix of contact capitation and fee-for-service remuneration with a focus on the second which incentives high frequencies with low consultation times {Bundesministerin für Gesundheit und Frauen, 2017 #144;Czypionka, 2015 #217;Redaèlli, 2015 #333;Bachner, 2018 #129}. | Financing type | Remuneration system of PC workforce |
| 6 | Coding of services and diagnosis | In 2030, there is a consistent and mandatory documentation of services and diagnoses (ICPC-2) in Austrian primary care. | Services are coded for remuneration by the social health insurance group. Coding of diagnoses is not mandatory {Bundesministerin für Gesundheit und Frauen, 2017 #144}. |  | Quality of diagnosis and treatment in PC |
| 7 | Quality assurance | In 2030, there exists a transparent measure for process and outcome in primary care (e.g. through quality indicators). | The Austrian Association for quality assurance and quality management in medicine is a limited company of the Austrian Medical Association and entrusted by law for the definition of criteria, evaluation, and registration regarding quality assurance. GPs are obliged to perform regular self-assessment of their practices. Further measures are voluntary {ÖQMED – Österreichische Gesellschaft für Qualitätssicherung & Qualitätsmanagement in der Medizin GmbH, #295}. | PC quality management infrastructure | PC quality management infrastructure |
| 8 | Countrywide optional patient registry in PC | In 2030, a countrywide optional patient registry is implemented in Austrian primary care. | There is no patient registry in PC {Kringos, 2013 #127}. | Patient lists | Longitudinal continuity of care |
| 9 | Advanced Practice Nurse (APN) in PC | In 2030, at least 500 advanced practice nurses (APN) work in Austrian primary care. | No national data available on the number of nurses and APNs working in PC {OECD, 2020 #308}  A study from Styria indicates that almost all practices (98%) employ a practice assistant but only 26% employ a graduate nurse {Korsatko, 2015 #235}. | Type of primary care practitioner | PC workforce supply and planning |
| 10 | Graduate nurses in PC | In 2030, at least 20% of all graduate nurses work in Austrian primary care. |  |  |  |
| 11 | Increasing number of general practitioners per population | In 2030, there is an average of one general practitioners per 2.000 inhabitants. | Declining proportion of GPs to all physicians from 2000 to 2019 {OECD, 2021 #318}  One of the lowest proportions of GPs compared to total physicians in the EU (Austria: 14%, EU: 21%) {OECD, 2021 #318}  In 2020, there were 44.5 GPs per 100,000 inhabitants {Österreichische Ärztekammer, 2020 #276}.  In 2016, on average one GP served a population of 2,334 inhabitants in 2016 {Stigler, 2020 #338}. |  | Geographic availability of PC services |
| 12 | Number of PCUs | In 2030, 225 primary health care units have been established in Austria. | 9 primary care units (end of 2018) {OECD, 2019 #317}  32 primary care units (mid 2021) {OECD, 2021 #318} |  |  |

Information on both years is mentioned if there were changes in the status in primary care between the survey at the end of 2019 and the submission of the paper in 2022.

APN: advanced practice nurse, GP: general practitioner, OECD: Organization for Economic Co-Operation and Development, PC: primary care, PCAT: primary care assessment tool, PCU: primary care unit

Literature

1. Information on both years is mentioned, if there were changes in the status in primary care between the survey at the end of 2019 and the submission of the paper in 2022. [↑](#footnote-ref-1)
