# Supplementary material for: Identifying key policy objectives for strong primary care: a cross-sectional study
Source: Prim Health Care Res Dev. 2023 Aug 14;24:e52. doi: 10.1017/S1463423623000403 (PMC10466205; doi:10.1017/S1463423623000403)
Supplement: Supplementary file 1 [file phcsup.zip › S1463423623000403sup003.docx]

**Additional file 2**

| **Characteristics of the participants** | **n** | **%** |
| --- | --- | --- |
| **Field of work** | | |
| Primary care: single-handed, group practices or PCUs | 38 | 24.8 |
| Community services and secondary care | 7 | 4.6 |
| Inpatient care (incl. outpatient departments and clinics, long-term care) | 22 | 14.4 |
| Science and research | 43 | 28.1 |
| Public administration (governmental organisations and social health insurance) | 33 | 21.6 |
| Associations and non-profit organisations | 10 | 6.5 |
| **Age** | | |
| < 30 years | 17 | 10.5 |
| 30 - 39 years | 33 | 20.4 |
| 40 - 49 years | 44 | 27.2 |
| 50 - 59 years | 49 | 30.2 |
| 60 - 69 years | 17 | 10.5 |
| ≥ 70 years | 2 | 1.2 |
| **Years in profession** | | |
| < 5 years | 18 | 11.0 |
| 5 - 10 years | 25 | 15.2 |
| 11 - 20 years | 42 | 25.6 |
| > 20 years | 76 | 46.3 |
| No health professional occupation | 3 | 1.8 |
| **Sex** | | |
| Female | 95 | 60.5 |
| Male | 62 | 39.5 |
| **Federal state of workplace** | | |
| Burgenland | 2 | 1.3 |
| Carinthia | 3 | 2.0 |
| Upper Austria | 15 | 9.8 |
| Lower Austria | 16 | 10.5 |
| Salzburg | 8 | 5.2 |
| Styria | 67 | 43.8 |
| Tyrol | 9 | 5.9 |
| Vorarlberg | 6 | 3.9 |
| Vienna | 27 | 17.6 |
